# Preferences for Postacute Care at Home vs Facilities

**DOI:** 10.1001/jamahealthforum.2024.0678

**Published:** 2024-04-26

**Authors:** Fangli Geng, Brian E. McGarry, Meredith B. Rosenthal, Jose R. Zubizarreta, Stephen C. Resch, David C. Grabowski

**Affiliations:** 1 Harvard University Graduate School of Arts and Sciences, Boston, Massachusetts; 2 Department of Health Care Policy, Harvard Medical School, Boston, Massachusetts; 3 Center for Health Decision Science, Harvard T.H. Chan School of Public Health, Boston, Massachusetts; 4 Department of Medicine, University of Rochester, Rochester, New York; 5 Department of Health Policy and Management, Harvard T.H. Chan School of Public Health, Boston, Massachusetts

## Abstract

**Importance:**

Two in 5 US hospital stays result in rehabilitative postacute care, typically through skilled nursing facilities (SNFs) or home health agencies (HHAs). However, a lack of clear guidelines and understanding of patient and caregiver preferences make it challenging to promote high-value patient-centered care.

**Objective:**

To assess preferences and willingness to pay for facility-based vs home-based postacute care among patients and caregivers, considering demographic variations.

**Design, Setting, and Participants:**

In September 2022, a nationally representative survey was conducted with participants 45 years or older. Using a discrete choice experiment, participants acting as patients or caregivers chose between facility-based and home-based postacute care that best met their preferences, needs, and family conditions. Survey weights were applied to generate nationally representative estimates.

**Main Outcomes and Measures:**

Preferences and willingness to pay for various attributes of postacute care settings were assessed, examining variation based on demographic factors, socioeconomic status, job security, and previous care experiences.

**Results:**

A total of 2077 adults were invited to participate in the survey; 1555 (74.9%) completed the survey. In the weighted sample, 52.9% of participants were women, 6.5% were Asian or Pacific Islander, 1.7% were American Indian or Alaska Native, 11.2% were Black or African American, 78.4% were White; the mean (SD) age was 62.6 (9.6) years; and there was a survey completion rate of 74.9%. Patients and caregivers showed a substantial willingness to pay for home-based and high-quality care. Patients and caregivers were willing to pay an additional $58.08 per day (95% CI, 45.32-70.83) and $45.54 per day (95% CI, 31.09-59.99) for HHA care compared with a shared SNF room, respectively. However, increased demands on caregiver time within an HHA scenario and socioeconomic challenges, such as insecure employment, shifted caregivers’ preferences toward facility-based care. There was a strong aversion to below average quality. To avoid below average SNF care, patients and caregivers were willing to pay $75.21 per day (95% CI, 61.68-88.75) and $79.10 per day (95% CI, 63.29-94.91) compared with average-quality care, respectively. Additionally, prior awareness and experience with postacute care was associated with willingness to pay for home-based care. No differences in preferences among patients and caregivers based on race, educational background, urban or rural residence, general health status, or housing type were observed.

**Conclusions and Relevance:**

The findings of this survey study underscore a prevailing preference for home-based postacute care, aligning with current policy trends. However, attention is warranted for disadvantaged groups who are potentially overlooked during the shift toward home-based care, particularly those facing caregiver constraints and socioeconomic hardships. Ensuring equitable support and improved quality measure tools are crucial for promoting patient-centric postacute care, with emphasis on addressing the needs of marginalized groups.

## Key Points

**Question:**

What are the postacute care preferences of patients and caregivers between home-based and facility-based settings?

**Findings:**

In this survey study and discrete choice experiment, patients and caregivers strongly preferred high-quality, home-based care; however, when caregiving burden exceeded 8 hours daily or participants faced socioeconomic challenges like job insecurity, caregivers’ preference shifted toward facility-based care, which were consistent across racial and educational backgrounds, and health statuses. Prior experience with postacute care was associated with an increased willingness to pay for home-based options.

**Meaning:**

The results of this survey study suggest that despite a predominant preference for home-based care, targeted support for disadvantaged groups facing caregiver constraints and socioeconomic hardships is essential to ensure equitable access and promote patient-centric postacute care.

## Teaser

This survey study examines preferences and willingness to pay for facility-based vs home-based postacute care among patients and caregivers.

## Introduction

Postacute care shapes immediate and long-term patient outcomes after injury, illness, or surgery.^1,2^ Two in 5 hospital stays are discharged to postacute care, which is mainly delivered by skilled nursing facilities (SNFs) and home health agencies (HHAs).^3^ Traditional Medicare spends nearly $60 billion annually on postacute care, accounting for 8.3% of total Medicare spending.^4^ As of 2020, 13 884 SNFs and 11 456 HHAs were in operation.^5,6^

Postacute care is the largest source of geographic variation in Medicare spending, presenting opportunities for quality improvement and cost efficiency.^7^ Although hospitalized patients typically receive discharge setting recommendations, the optimal setting for heterogeneous patients remains ambiguous. Furthermore, quality measures, as reported by the US Centers for Medicare & Medicaid Services (CMS) Nursing Home and Home Health Care Compare tools, do not typically facilitate direct comparisons between SNFs and HHAs. Consequently, patients and caregivers often hastily base postacute care decisions on prior experiences, perceptions, preferences, and trade-offs concerning quality, cost, and location. This situation is exacerbated by insufficient comparative postacute care quality information, potentially impeding informed decisions.^8^

Efforts have been made to bolster consumer decision-making and discharge planning. The Improving Medicare Post-Acute Care Transformation Act of 2014 mandates standardized care quality reporting and patient assessment metrics across postacute care settings.^9^ Additionally, the 2019 CMS Hospital Discharge Rule requires hospitals to provide patients with a list of postacute care options containing quality and resource-use measures.^10^ However, hospitals, which substantially contribute to patients’ discharge decision-making processes, also have their own incentives and may lack a comprehensive understanding of the preferences of diverse patient and caregiver groups.

A growing policy interest exists in shifting patient care from institutional toward home-based postacute settings, which is exemplified by the growth in alternative payment models within traditional Medicare and the expansion of beneficiaries in Medicare Advantage, that is typically associated with reduced postacute care use.^11,12,13^ The COVID-19 pandemic further accelerated this trend from SNFs to HHAs.^14^ However, questions persist regarding whether these developments align with actual consumer preferences and desired care settings.

A substantial gap exists in the literature concerning the systematic quantification of postacute care consumer preferences and economic value determinants. Home-based care may inadvertently shift a substantial caregiving burden onto families, a dimension seldom factored into current cost implications.

Our research endeavored to bridge this knowledge gap by quantifying patients’ and caregivers’ preferences and willingness to pay for different features of postacute care settings, focusing specifically on SNFs and HHAs. The application of a discrete choice experiment (DCE) allowed us to observe preferences that may not be fully captured through clinical decisions. Decisions made in the clinical setting reflect postacute care availability, Medicare policies, resources, availability of family, and many other observed and unobserved factors. Due to these factors, clinical postacute care choices do not always reflect actual preferences.^15^ Additionally, preferences across patients and caregivers are difficult to discern from clinical decisions. By leveraging the DCE methods, which have been used in numerous health policy and clinical contexts,^16,17^ we aimed to closely simulate the actual decision-making process, considering resource constraints and other factors.

## Methods

This study followed the Strengthening the Reporting of Observational Studies in Epidemiology (STROBE) reporting guideline for survey studies and received approval from the Harvard University institutional review board.^18^ The protocol of the RAND American Life Panel (ALP), for which the survey was conducted, undergoes annual review by the RAND institutional review board. Study participants provided written informed consent through the ALP’s annual consent statement.

### Data Sources

We conducted a DCE to estimate group-level preferences and assess the collective willingness to pay for specific postacute care attributes. Our survey was conducted through the ALP, a nationally representative internet-based panel operated by the RAND Corporation. The survey ran from August 4 to September 9, 2022, achieving a 74.9% completion rate from 2077 invited panel members 45 years or older. We targeted this age group because they encompass most discharges to postacute care and are often the primary postacute care decision-makers for themselves and their families. The technical specifics of the ALP and sampling method have been detailed elsewhere.^19^ To ensure external validity, we confirmed that key demographic variables in our weighted sample were similar to US Census Bureau data from 2022 (eTable 1 in Supplement 1).

### Survey Design

Participants were presented with 8 hypothetical choice scenarios (eMethods 1 in Supplement 1). In each scenario, they were presented with the options of an SNF and an HHA and asked, “Which option would you choose?” to best meet their preferences, needs, and family conditions. Attributes defining these choices were identified through a literature review and expert consultation to ensure the relevance and importance to respondents, as well as policy and clinical decision-making. Our experiment considered 6 attributes: travel time from home (10 minutes, 30 minutes, and 90 minutes for SNFs and no travel time for HHAs), room type (private room or semiprivate room for SNFs and stay at home for HHAs), overall quality rating of the facility/agency (much above average, above average, average, and below average for SNFs and HHAs), daily caregiving hours required from a family member (0 hours, 1 hour, and 3 hours for SNFs and 2 hours, 5 hours, and 9 hours for HHAs), recovery period (20 days, 30 days, and 45 days for SNFs and HHAs), and out-of-pocket costs ($250, $500, $1500, and $3000 for SNFs and HHAs).

To discern preferences between patients and caregivers, participants were randomly divided into 2 groups: those deciding for themselves as patients and those deciding as primary caregivers for a 70-year-old family member. Participants were prompted to envision postacute care needs following a hospital stay for a hip replacement or stroke. These conditions were chosen because they are among the most common reasons for postacute care discharge and require different therapy regimens and recovery timelines. Moreover, patients with these conditions are frequently seen in institutional and home-based postacute care.

Descriptions of these health conditions and their severities were constructed so that a participant’s decision between an SNF or HHA would be predominantly associated with their personal preferences rather than any clinical judgment. To mimic a clinical decision-making process, we did not incorporate an opt-out or status-quo option in our study.

To ensure the clarity and accuracy of survey instructions and attributes, we undertook survey comprehension testing and 2 pilot studies before administering the full survey. This process confirmed that our survey instructions and attributes aligned with participants’ understanding (eMethods 2 in Supplement 1). To assess participants’ engagement and interest in the final survey content, we included a specific question of “How interesting are the questions in the survey?” with possible responses ranging from “very interesting” to “very uninteresting.”

### Discrete Choice Experiment Design

We used a labeled, D-efficient design for the DCE with no interaction effects, allowing for alternative-specific parameter estimates for facility/agency quality ratings (eMethods 2 in Supplement 1). The design was created using the Ngene software, version 1.1 (ChoiceMetrics). We used repeated questions with varying choice attributes that comprised a total of 48 choice tasks divided into 6 blocks. Each participant was assigned to 1 block containing distinct choice tasks. The design adhered to the ISPOR good research practice and experimental guidelines of the conjoint analysis task force.^20,21^

### Additional Questions

The ALP annual survey provided demographic information, employment status, living conditions, educational background, income level, health insurance type, and self-rated general health. We added questions about participants’ previous experience with SNFs and HHAs and their support system for postacute care (for the patient perspective survey) or their job flexibility and security (for the caregiver perspective survey).

### Statistical Analysis

We evaluated 3 models: binary logit, alternative-specific logit, and mixed logit models (eMethods 3 in Supplement 1). Based on the Akaike Information Criterion and Bayesian Information Criterion (eTable 2 in Supplement 1), our primary results were based on a mixed logit model that incorporated alternative-specific coefficients for quality of care in SNFs and HHAs (eTable 3 in Supplement 1).^22^ The calculations for willingness to pay are provided in eMethods 4 in Supplement 1.

We accounted for the 8 observations per participant by allowing for dependence in the error terms among observations from the same individual, as these observations may share unobserved characteristics. Survey weights were applied to generate nationally representative estimates. We computed confidence intervals of the willingness to pay for each attribute using the Delta method. Statistical analyses were conducted using the PandasBiogeme package 3.2.10 (2022) in Python.^23^

To understand the demographic and socioeconomic factors that may be associated with care preferences, we adjusted our main effect models using unique parameters (interaction terms) for various groups. These parameters included sex, marital status, living arrangements, urban or rural residence, employment status, type of housing, self-reported race, general health, previous experience with SNFs and HHAs, age, family income, and education. In the case of caregiver respondents, we also studied their work flexibility and job security.

## Results

### Descriptive Analysis

In the weighted sample, 52.9% were women with a mean (SD) age of 62.6 (9.6) years. A total of 35.5% held a Bachelor degree or higher. The weighted sample consisted of 2.2% Asian or Pacific Islander respondents, 1.7% American Indian or Alaska Native, 11.2% African American, 78.4% White, and 6.5% of other categories (may comprise people of multiracial backgrounds or those who identify with a racial group not explicitly listed in the categories of the survey). The average household size was 2.53 persons, and 76.5% resided in single-family houses (Table). Further participant details are available in eTable 1 in Supplement 1. We received 787 responses from the patient perspective and 768 from caregivers. Regarding participant engagement, 82.2% of respondents found the survey content to be either very interesting or interesting, suggesting a high level of participant engagement (eFigure in Supplement 1).^24^ Survey completers had comparable demographic characteristics with those who did not complete the survey, bolstering the validity of our survey (eTable 4 in Supplement 1).

**Table.** Weighted Characteristics of 1555 Study Participants Who Completed the Survey Who Were Representative of a National Sample^a^

Characteristic	Weighted %
Female	52.9
Male	47.1
Age, mean (SD) y	62.6 (9.6)
Education	
Less than or some high school with no diploma	7.3
High school graduate or equivalent	32.2
Some college, no degree	15.0
Associate degree	10.2
Bachelor’s degree and higher	35.3
Race	
Asian or Pacific Islander	6.5
American Indian or Alaska Native	1.7
Black/African American	11.2
White	78.4
Other^b^	6.5
Household size (persons per household), mean	2.53
House type	
Single-family house	76.5
Building with apartments	19.1
Mobile home, boat, recreational vehicle, van	4.4
Annual household income, $	
<19 999	12.4
20 000 to 39 999	18.2
40 000 to 74 999	28.5
75 000 to 124 999	20.6
125 000 to 199 999	14.0
≥200 000	6.3
Urban/rural	
Small to midsize city or large city with a population ≥50 000	73.8
Rural or small town, population <50 000	26.2
General health	
Excellent	10.3
Very good	37.8
Good	34.1
Fair	15.1
Poor	2.7

^a^ Analysis of data from the online survey conducted with the American Life Panel. Percentages might not add to 100 because of rounding. Statistics have been reweighted for nationally representativeness. The survey questions concerning demographic characteristics were modeled after those in the Current Population Survey conducted by the US Census Bureau for the US Bureau of Labor Statistics. Participants were asked to complete the survey, which included a range of demographic questions on a quarterly basis.

^b^ Other may comprise people of multiracial backgrounds or those who identify with a racial group not explicitly listed in the categories of the survey.

In the weighted sample, prior experience with SNFs and HHAs was common among participants (eTable 5 in Supplement 1). Specifically, 33.1% of patients and 29.4% of caregivers had cared for a family member in a nursing home, while 39.4% of patients and 40.5% of caregivers had taken care of a family member with HHA care. Nevertheless, 33.3% of patients and 29.6% of caregivers reported no direct experience with or did not know much about SNFs; for HHAs, these figures were 51.4% and 51.5%, respectively.

Regarding the postacute care decision process, patients (59.8%) and caregivers (55.3%) favored joint decision-making. Meanwhile, 29.2% of patients and 33.3% of caregiver favored decisions primarily based on the patient’s preference. Only 11.0% of patients and 11.4% of caregivers indicated relying mainly on the caregiver’s preferences.

### Statistical Analysis

**Figure 1.**
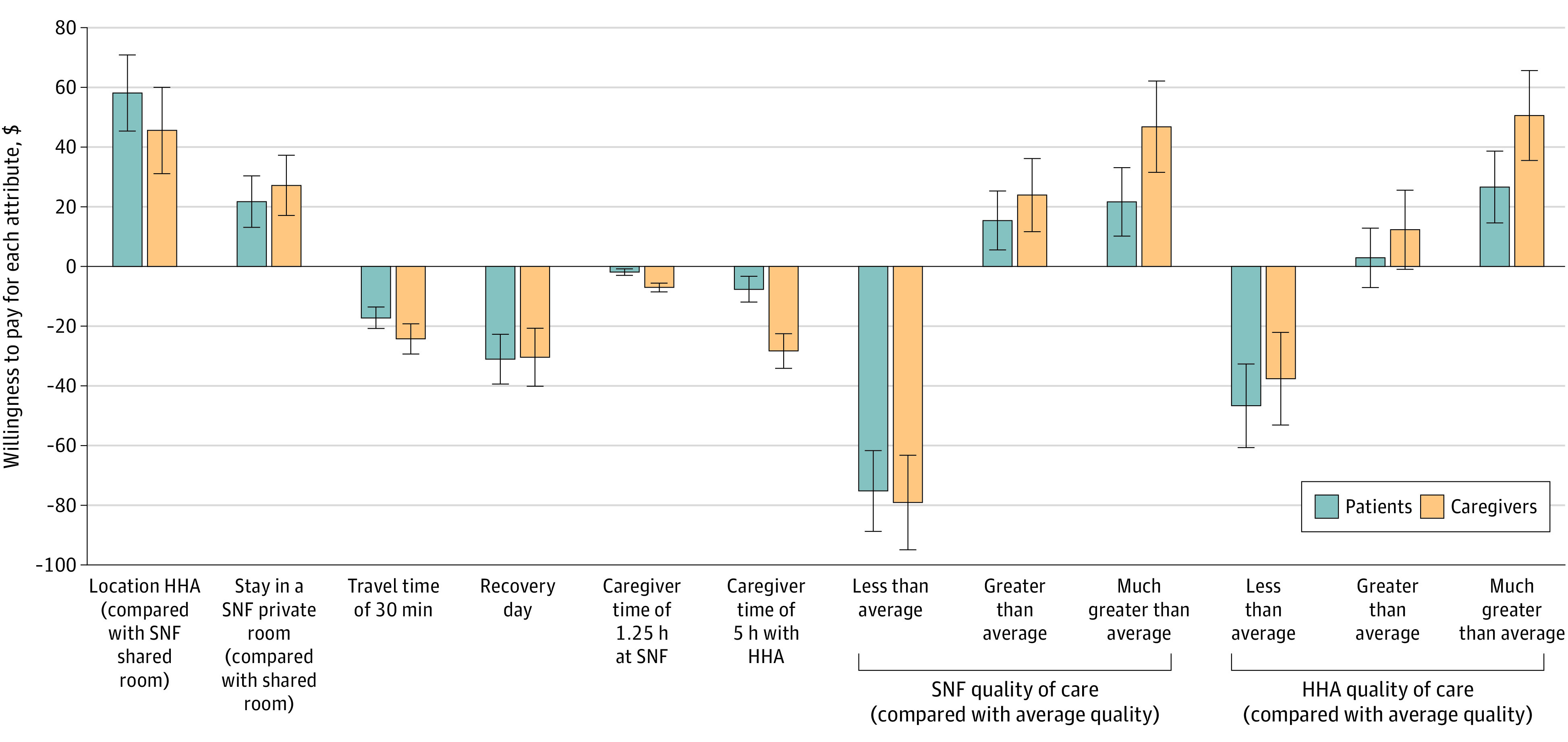
Patients’ and Caregivers’ Willingness to Pay for Each Postacute Care Attribute per Day Analysis of data from the online survey conducted with the American Life Panel. Model output from the alternative-specific model (eTable 2 in Supplement 1). The error bars represent the 95% CIs of the point estimates for each willingness-to-pay estimate. HHA indicates home health agency; SNF, skilled nursing facility.

The patient and caregiver groups had a higher willingness to pay for at-home care, superior quality, decreased travel times, shorter recovery period, and less caregiver involvement (Figure 1). Among all attributes, the avoidance of below average care and receiving care at home proved highly significant to patients and caregivers. They also moderately prioritized high-quality care, rapid recovery time, private room accommodation, and reduced travel time. For patients, caregiver time appeared to be less of a burden, yet when such time increased significantly, its importance grew for caregivers.

On average, respondents were willing to pay an extra $51.81 per day for at-home care ($58.08 for patients and $45.54 for caregivers) compared with staying in a shared room at an SNF. Also, they were willing to pay an additional $24.44 per day for private room accommodation at an SNF ($21.74 more for patients and $27.15 more for caregivers) compared with a shared room. Decreasing daily travel time by 30 minutes was valued at $20.50 per day ($17.23 for patients and $24.29 for caregivers). Shortening recovery time by 1 day was valued at $30.75.

Considering caregiver involvement, an hour saved for patient care was valued at $1.53 by patients and $5.66 by caregivers. As such, when patients stayed at an SNF and caregivers provided 1.25 hours of daily care, respondents were willing to pay an average of $4.50 to save caregiver time ($1.91 for patients and $7.06 for caregivers).^25^ This willingness to pay increased to $7.63 for patients and $28.32 for caregivers when the patients received at-home care from an HHA requiring 5 hours of daily caregiver input.

Our data highlighted a unanimous preference for higher-quality care with a pronounced aversion to below average care. Caregivers were more inclined to pay a premium for better care for patients than the patients themselves. For instance, to avoid below average care at SNFs, respondents were willing to pay an average of $75.21 (patients) and $79.10 (caregivers) per day for average-quality SNF care. Similar trends were observed regarding HHAs. Compared with average-quality HHA care, both groups were willing to pay $46.65 (patients) and $37.58 (caregivers) per day to avoid below average care and $26.59 (patients) and $50.55 (caregivers) per day for much above average care.

**Figure 2.**
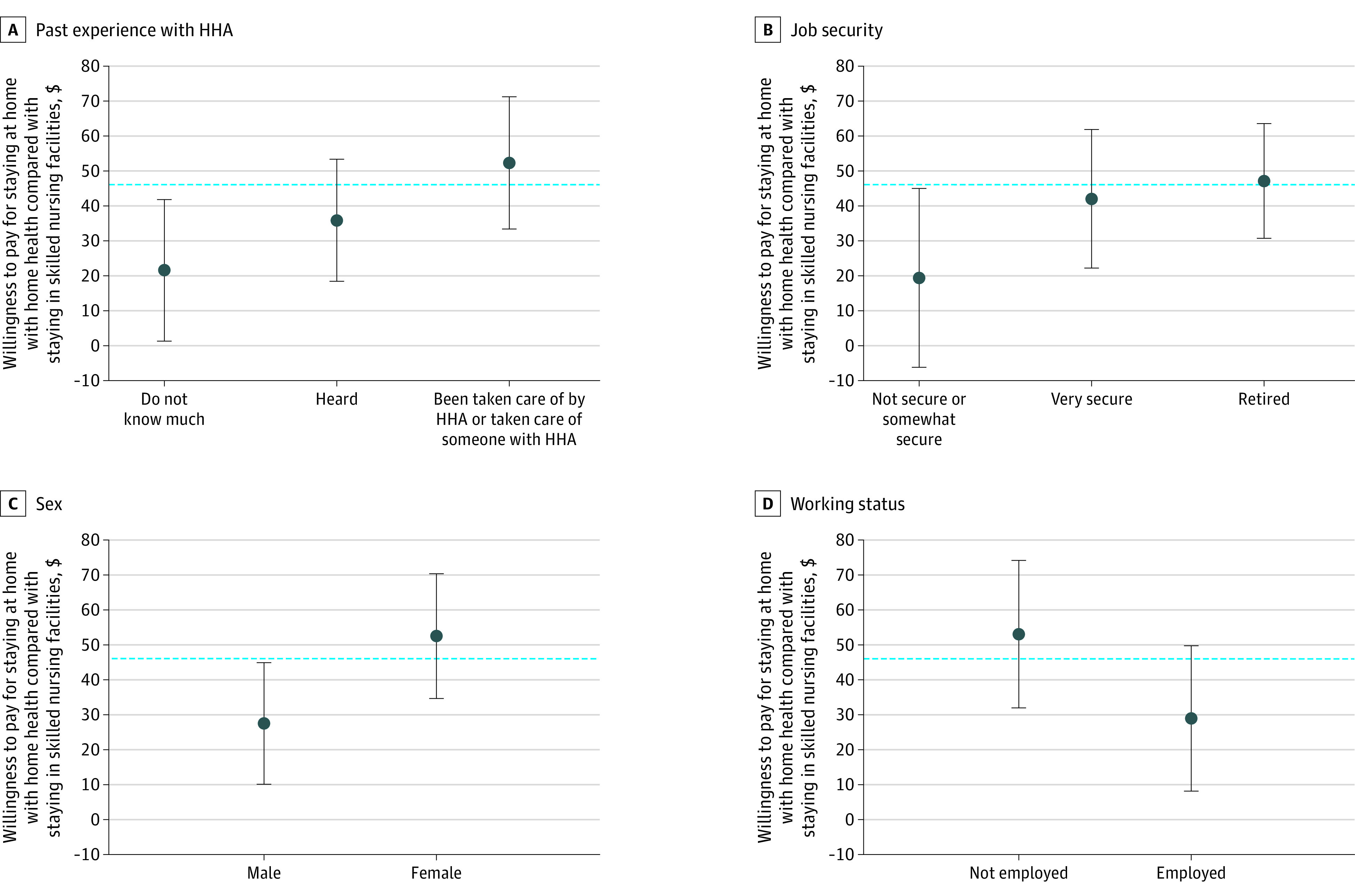
Willingness to Pay for Home Health vs Skilled Nursing Facilities (SNFs) by Caregiver Characteristics Analysis of data from the online survey conducted with the American Life Panel. The error bars represent the 95% CIs of the point estimates for each willingness-to-pay estimate. The blue dotted line represents the national average for how much caregivers are willing to pay for patients to stay at home with home health compared with staying in SNFs. For the details of the alternative-specific constant models, see eMethods 3 in Supplement 1. HHA indicates home health agency.

Our examination of various socioeconomic factors revealed that factors such as job security, employment status, previous experience with HHAs and SNFs, and being female were all significantly associated with caregivers’ willingness to pay for home-based postacute care vs SNFs (Figure 2). Caregivers with insecure jobs were only willing to pay an additional $19.37 per day, while those with more job security were willing to pay $42.00 per day. Similarly, employed caregivers indicated a willingness to pay $28.94 more for home-based care compared with $53.03 among unemployed caregivers. On average, female caregivers demonstrated a willingness to pay an additional $52.50 for patients to receive home-based care compared with $27.53 among male respondents. Caregivers who had personal experience with HHAs indicated the highest willingness to pay at $52.28 more per day for the HHA option compared with staying at a shared room in SNFs.

**Figure 3.**
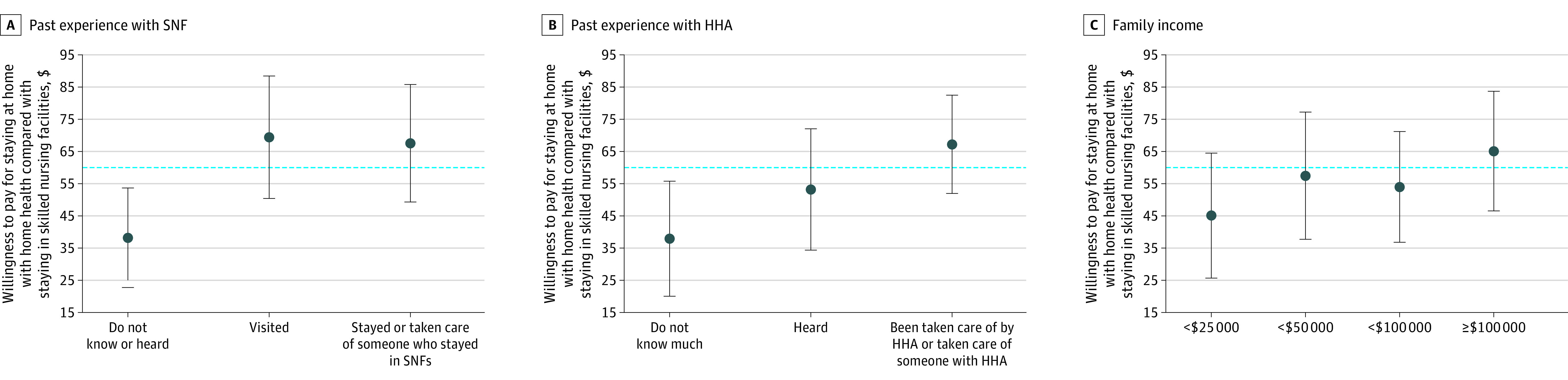
Willingness to Pay for Home Health vs Skilled Nursing Facilities (SNFs) by Patient Characteristics Analysis of data from the online survey conducted with the American Life Panel. The error bars represent the 95% CIs of the point estimates for each willingness-to-pay estimate. The blue dotted line represents the national average for how much patients are willing to pay for staying at home with home health compared with staying in SNFs. For the details of the alternative-specific constant models, see eMethods 3 in Supplement 1. HHA indicates home health agency.

In the case of the patient group, their prior experience with SNFs and HHAs, as well as their family income, was significantly associated with their preferences concerning whether to receive care at home through HHAs or at an SNF (Figure 3). Additionally, we observed no differences in preferences among patients and caregivers based on race, educational background, urban or rural residence, general health status, or housing type.

## Discussion

This survey study highlighted patients’ and caregivers’ preferences for home-based and high-quality postacute care. Secondary considerations include travel time, recovery duration, and a private room. The preferences between patients and caregivers diverged significantly when caregiver time exceeded 5 hours daily. Associated factors shaping postacute preferences included socioeconomic status, employment security, and previous experiences with HHAs and SNFs.

These results underscore that the current policy shift toward home-based postacute care is largely consistent with patient and caregiver preferences. However, when caregiving demands escalate to the equivalent of a full-time job (exceeding 8 hours per day), caregivers shift to favoring SNFs. This trend was amplified among socioeconomically disadvantaged groups, such as those in insecure jobs. Despite a clear preference for home-based care within these groups, the added caregiving burden can overshadow its benefits, underlining the need for policies that address these disparities. Additionally, female caregivers displayed a higher willingness to pay for patients’ home-based care, likely in part due to the disproportionate caregiving burden they face.

Given patients’ and caregivers’ willingness to pay an extra $51.81 per day for home-based care, our financial and social systems must ensure adequate support of home-based care across diverse socioeconomic contexts. The Biden Administration recently announced a package of reforms to improve care, including providing more support to family caregivers during the hospital discharge planning process.^26^ Those reforms provide an opportunity to address the underlying complexities identified in our study, ensuring a more equitable distribution of care that reflects patient preferences and caregiver capacities.

The prioritization of quality by patients and caregivers supports the major investments by CMS in the Care Compare websites. However, the variability in the quality measures across the HHA and SNF Care Compare sites is a major limitation. For example, the HHA tool contains information on patient satisfaction, while the SNF tool does not. Another issue is that many patients are unaware of these tools, and discharge planners do not typically use them in assisting patients.^3,27^ Although the 2019 CMS Hospital Discharge Rule requires the discharge planner to assist in selecting a postacute care clinician or health care facility by sharing key performance data, it is unclear whether this rule is currently being enforced and followed. We would encourage increased oversight of the postacute discharge process.

In support of improved discharge planning, those with prior experience or familiarity with HHAs and SNFs demonstrated a stronger preference for home-based postacute care. This observation highlights the importance of patient and caregiver education about postacute care options. Proactive measures, such as collecting comprehensive postacute quality information and developing decision aids, may significantly guide preference-aligned choices.

Alternative payment models in traditional Medicare and Medicare Advantage plans have shifted postacute care away from institutional settings. The insights from our study can potentially help these value-based efforts deliver postacute services that closely meet the needs and preferences of patients and caregivers. Models that encourage greater HHA use should account for the financial and nonfinancial costs placed on caregivers. Because home-based care places a financial burden on family caregivers, we need to ensure that these policies do not increase disparities in care for disadvantaged groups.

### Limitations

Our study had limitations. The data drew from a national sample of individuals 45 years and older, with a specific focus on hip replacement and stroke. Consequently, the generalizability of our findings to different age groups and other medical conditions may be limited. Nonetheless, our estimates display good concordance with available clinical price data. On average, we found that patients and caregivers were willing to pay an additional $25 per day for a private room, which aligns closely with the $27 per day difference between the cost of private and semiprivate rooms for private payers obtained from secret shopper data.^28^ Additionally, our experiment considered the SNF and HHA options, which encompass most postacute care discharges. However, we acknowledge that some patients do receive care in other settings, such as inpatient rehabilitation facilities.

## Conclusions

The results of this survey study underscore the preference for home-based postacute care among patients and caregivers, which is consistent with the current policy direction. Nevertheless, our research emphasizes the need to consider factors such as caregiver availability and job security, family income levels, and previous experiences with SNFs and HHAs. Furthermore, our findings demonstrate a strong preference for postacute quality. These insights may offer valuable guidance to policymakers, health care clinician and networks, and insurers as they strive to design and deliver high-value, patient-centered postacute care, with a particular focus on the needs of disadvantaged groups.

## Notes

Supplement 1. eMethods 1. An example of survey eMethods 2. Technical details about the experimental design of our survey eMethods 3. Model specifications eTable 1. Additional Weighted Characteristics of Study Participants Who Completed the Survey (N=1,555), Representative of a National Sample eTable 2. Main-effect model performance comparison eTable 3. Regression analysis results for the best performing main-effect model - mixed logit model with alternative specific coefficients for quality of care and recovery time. eMethods 4. Calculation of Willingness-to-Pay (WTP) Estimates and the underlying assumptions for WTP estimates. eFigure 1. Participant Engagement: Responses to Survey Interest Query eTable 4. Descriptive Statistics of Respondent Characteristics, Comparing Unweighted Data Between Survey Completers and Non-Completers eTable 5. Prior experience with skilled nursing facilities and home health care and post-acute care decision-making

Supplement 2. Data sharing statement
