# Supplementary material for: Preferences for Postacute Care at Home vs Facilities
Source: JAMA Health Forum. 2024 Apr 26;5(4):e240678. doi: 10.1001/jamahealthforum.2024.0678 (PMC11065156; doi:10.1001/jamahealthforum.2024.0678)
Supplement: Supplement 1. — eMethods 1. An example of survey eMethods 2. Technical details about the experimental design of our survey eMethods 3. Model specifications eTable 1. Additional Weighted Characteristics of Study Participants Who Completed the Survey (N=1,555), Representative of a National Sample eTable 2. Main-effect model performance comparison eTable 3. Regression analysis results for the best performing main-effect model - mixed logit model with alternative specific coefficients for quality of care and recovery time. eMethods 4. Calculation of Willingness-to-Pay (WTP) Estimates and the underlying assumptions for WTP estimates. eFigure 1. Participant Engagement: Responses to Survey Interest Query eTable 4. Descriptive Statistics of Respondent Characteristics, Comparing Unweighted Data Between Survey Completers and Non-Completers eTable 5. Prior experience with skilled nursing facilities and home health care and post-acute care decision-making [file jamahealthforum-e240678-s001.pdf]

Supplemental Online Content

Geng F, McGarry BE, Rosenthal MB, Zubizarreta JR, Resch SC, Grabowski DC. Preferences for postacute care at home vs facilities. *JAMA Health Forum*. 2024;5(4):e240678. doi:10.1001/jamahealthforum.2024.0678

eMethods 1. An example of survey

eMethods 2. Technical details about the experimental design of our survey

eMethods 3. Model specifications

eTable 1. Additional Weighted Characteristics of Study Participants Who Completed the Survey (N=1,555), Representative of a National Sample

eTable 2. Main-effect model performance comparison

eTable 3. Regression analysis results for the best performing main-effect model - mixed logit model with alternative specific coefficients for quality of care and recovery time.

eMethods 4. Calculation of Willingness-to-Pay (WTP) Estimates and the underlying assumptions for WTP estimates.

eFigure 1. Participant Engagement: Responses to Survey Interest Query

eTable 4. Descriptive Statistics of Respondent Characteristics, Comparing Unweighted Data Between Survey Completers and Non-Completers

eTable 5. Prior experience with skilled nursing facilities and home health care and post-acute care decision-making

This supplemental material has been provided by the authors to give readers additional information about their work.

© 2024 Geng F et al. *JAMA Health Forum.*

eMethod 1. An example of Survey

Survey: (alternative 1) Preferences for Post-Hospital Care as a Patient

Introduction

Thank you for your participation in this survey on **preferences for post-hospital** services. Through this survey, you will help researchers at Harvard Medical School learn more about **how people choose facilities and services following a hospital stay**. Your careful completion of the survey will help to reshape health care policy based on people's preferences.
Post-hospital care refers to the services people receive after a stay in a hospital. These services **help patients recover and rehabilitate** from a surgery or injury after they are stable enough to be discharged from the hospital. Conditions that typically require post-hospital care include hip fracture, stroke, and heart failure. Depending on how much care is needed, treatment may include a stay in a skilled nursing facility (also known as a nursing home), or care provided at home by a home health agency.
The survey will take about 5 minutes to complete. Thank you for your participation!

Choice Experiment

[Vignette by Condition -*Hip Fracture/Stroke* – randomized]

For the following set of **8 choice tasks**, imagine that you were **hospitalized for [*hip fracture/stroke*]** and are about to be discharged from the hospital. Your doctor recommends **a recovery period of post-hospital care** to ensure a successful recovery period. Your doctor tells you that, from a clinical perspective, you are a good fit for either care in a skilled nursing facility (i.e., a nursing home) or care at home through visits from home health aides, nurses and physical, occupational [*therapists (for hip fracture) /, and speech therapists (for stroke)*] who work for a home health agency].

You will need some **assistance with daily tasks** like bathing, getting dressed, [*using the bathroom (for hip fracture) / using the bathroom, and eating (for stroke)*] during the first week and gradually recover to perform those activities without assistance by the end of the recovery period. You will also need **[*physical and occupational therapy sessions (for hip fracture)/physical, occupational and speech therapy sessions (for stroke)*]** to help you recover your strength, balance, and ability to move your body, perform activities of [*daily living (for hip fracture) / daily living and communication (for stroke)*]. The frequency of the therapy sessions will be provided based on your health conditions. You can assume that at the end of the recovery period, you will no longer need any specialized care for your [*hip injury/stroke*] condition.

You have a choice between receiving care either 1) in **a skilled nursing facility (or nursing home).** The primary people assisting with your daily living are **nurse aides**, **or 2) in your home with visits from a home health agency**. The primary people assisting with your daily living are **family members and home health aides**.

© 2024 Geng F et al. *JAMA Health Forum.*

Please select the service that **best meets your preferences, needs and family conditions**. Please assume all other aspects of the choices (such as your health conditions) are the same except for the attributes listed in the questions. Each choice set represents a new decision and is unrelated to other decisions.

Choice set 1 of 8

**Options**	**Choice A<br>Skilled nursing facility**	**Choice B<br>Home with home health agency**
Travel time from your home	30min	No travel time
Room type	Stay in a private room	Stay at home
Overall quality rating of the facility/agency*	much above average	average
Time that your primary caregiver from family need to spend taking care of you per day	3 hours	5 hours
Recovery period	45 days	20 days
Total amount you need to pay for post-hospital care after insurance coverage	$3000	$1500
**Which option would you choose?**	**Choice A** ▯	**Choice B** ▯

***Overall quality rating** represents overall quality performance in three areas: **health inspections, staffing, and quality measures**. "Average" for a nursing home means that the nursing home has average quality among all the nursing homes. "Average" for a home health agency means that the home health agency has average quality among all the home health agencies.

(We alternated the positions for Choice A and Choice B across different respondents to avoid position effects for our labeled design.)

© 2024 Geng F et al. *JAMA Health Forum.*

Levels for each attribute:

Options	Choice A	Choice B
Post-hospital care type	Care at a skilled nursing facility (or nursing home)	Care at home with visits from home health agency
Travel time from your home	-10min<br>-30min<br>-90min	-No travel time
Total amount you need to pay for post-hospital care after insurance coverage	-$250<br>-$500<br>-$1500<br>-$3000	-$250<br>-$500<br>-$1500<br>-$3000
Overall quality rating of the facility/agency	- much above average<br>- above average<br>- average<br>- below average	- much above average<br>- above average<br>- average<br>- below average
Recovery period	-20 days<br>-30 days<br>-45 days	-20 days<br>-30 days<br>-45 days
Room type	- Stay in a private room<br>- Stay in a shared room with another patient	- Stay at home
Primary person assisting with daily living	nurse aides at skilled nursing facility	family members and home health aides
Time that your primary caregiver from family spends to take care of you per day	-0 hour<br>-1 hour<br>-3 hour	-2 hour<br>-5 hours<br>-9 hours

Background information and experience with SNFs and HHAs

Q1. What is your experience with skilled nursing facilities (also known as nursing homes)?

1. I have stayed in at least one skilled nursing facility.
2. I have taken care of at least one family member who has stayed in a skilled nursing facility.
3. I have visited at least one skilled nursing facility.
4. I have never had direct experience with a skilled nursing facility but have heard about people's experiences in skilled nursing facilities.
5. I don't know much about skilled nursing facilities.

Q2. What is your experience with home health agencies?

1. I have been taken care of by at least one home health agency.

© 2024 Geng F et al. *JAMA Health Forum.*

2. I have taken care of at least one family member who has been taken care of by at least one home health agency
3. I have never had direct experience with a home health agency but have heard about people’s experiences with home health agencies.
4. I don’t know much about home health agencies.

Q3. Do you have a friend or family member who currently lives with you, could temporarily live with you, or could make daily visits to your home to help take care of you while you recover?

1. Yes.
2. No.

Q4. If you and your primary caregiver have different preferences for the type of post-hospital care you receive, how will you decide where to receive post-hospital care?

1. I will decide mostly based on my preference and my caregiver will respect my decision.
2. I will discuss with my primary caregiver and decide based on both of our preferences.
3. I will decide mostly based on my caregiver’s preference as I will rely on him/her to provide the needed care.

© 2024 Geng F et al. *JAMA Health Forum.*

Survey: (alternative 2) Preferences for Post-Hospital Care as a Caregiver

Introduction

Thank you for your participation in this survey on **preferences for post-hospital care** services. Through this survey, you will help researchers at Harvard Medical School learn more about **how people choose post-hospital care facilities and services**. Your careful completion of the survey will help to reshape health care policy based on people's preferences.
Post-hospital care refers to the services people receive after a stay in a hospital. These services **help patients recover and rehabilitate** from a surgery or injury after they are stable enough to be discharged from the hospital. Conditions that typically require post-hospital care include hip fracture, stroke, and heart failure. Depending on how much care is needed, treatment may include a stay in a skilled nursing facility (also known as a nursing home), or care provided at home by a home health agency.
The survey will take about 5 minutes to complete. Thank you for your participation!

Choice Experiment

[Vignette by Condition and by gender-Hip Fracture/Stroke – randomized]

For the following set of **8 choice tasks**, imagine that your **70-year-old close family member** was **hospitalized for [*hip fracture/stroke*]** and is about to be discharged from the hospital. His or her doctor recommends **a recovery period of post-hospital care** to ensure a successful recovery period. The doctor tells you that, from a clinical perspective, he or she is a good fit for either care in a skilled nursing facility (i.e., a nursing home) or care at home through visits from home health aides, nurses and physical, occupational [*therapists (for hip fracture) /, and speech therapists (for stroke)*] who work for a home health agency].

During the recovery, **you will be the primary caregiver** for your family member. Your family member will need some **assistance with daily tasks** like bathing, getting dressed, [*using the bathroom (for hip fracture) / using the bathroom, and eating (for stroke)*] during the first week and gradually recover to perform those activities without assistance by the end of the recovery period. He or she will also need [***physical and occupational therapy sessions (for hip fracture)/physical, occupational and speech therapy sessions (for stroke)***] to help recover his or her strength, balance, and ability to move the body, perform activities of [*daily living (for hip fracture) / daily living and communicate (for stroke)*]. The frequency of the therapy sessions will be provided based on his or her health conditions. You can assume that at the end of the recovery period, he or she will no longer need any specialized care for his or her [*hip injury/stroke*] condition.

As the primary caregiver, you will help your family member make a choice between receiving care either 1) in **a skilled nursing facility (or nursing home).** The primary people assisting with your family member's daily living are **nurse aides**, **or 2) in his or her home with visits from a home health agency.** The primary people assisting with daily living are **home health aides and you**.

© 2024 Geng F et al. *JAMA Health Forum.*

Please select the service that **best meets your family's preferences, needs, and conditions**. Please assume all other aspects of the choices (such as the health conditions of your family member) are the same except for the attributes listed in the questions. Each choice set represents a new decision and is unrelated to other decisions.

Choice set 1 of 8

**Options**	**Choice A<br>Skilled nursing facility**	**Choice B<br>Home with home health agency**
Travel time from your home	30min	No travel time
Room type	Stay in a private room	Stay at home
Overall quality rating of the facility/agency*	much above average	average
**Time that you need to spend taking care of your family member per day**	3 hours	5 hours
Recovery period	45 days	20 days
Total amount your family member needs to pay for post-hospital care<br>after insurance coverage	$3000	$1500
**Which option would you choose?**	**Choice A** ☐	**Choice B** ☐

***Overall quality rating** represents overall quality performance in three areas: **health inspections, staffing, and quality measures**. "Average" for a nursing home means that the nursing home has average quality among all the nursing homes. "Average" for a home health agency means that the home health agency has average quality among all the home health agencies.

(We alternated the positions for Choice A and Choice B across different respondents to avoid position effects for our labeled design.)

© 2024 Geng F et al. *JAMA Health Forum.*

Levels for each attribute:

Options	Choice A	Choice B
Post-hospital care type	Care at a skilled nursing facility (or nursing home)	Care at home with visits from home health agency
Travel time from your home	-10min<br>-30min<br>-90min	-No travel time
Total amount your family member needs to pay for post-hospital care after insurance coverage	-$250<br>-$500<br>-$1500<br>-$3000	-$250<br>-$500<br>-$1500<br>-$3000
Overall quality rating of the facility/agency	- much above average<br>- above average<br>- average<br>- below average	- much above average<br>- above average<br>- average<br>- below average
Recovery period	-20 days<br>-30 days<br>-45 days	-20 days<br>-30 days<br>-45 days
Room type	- Stay in a private room<br>- Stay in a shared room with another patient	- Stay at home
Primary person assisting with daily living	Nurse aides at skilled nursing facility	You and home health aides
Time that you need to spend taking care of your family member per day	-0 hour<br>-1 hour<br>-3 hours	-2 hour<br>-5 hours<br>-9 hours

Background information and experience with SNFs and HHAs

Q1. What is your experience with skilled nursing facilities (also known as nursing homes)?

1. I have stayed in at least one skilled nursing facility.
2. I have taken care of at least one family member who has stayed in a skilled nursing facility.
3. I have visited at least one skilled nursing facility.
4. I have never had direct experience with a skilled nursing facility but have heard about people's experiences in skilled nursing facilities.
5. I don't know much about skilled nursing facilities.

Q2. What is your experience with home health agencies? (visiting services at home)

© 2024 Geng F et al. *JAMA Health Forum.*

1. I have been taken care of by at least one home health agency.
2. I have taken care of at least one family member who has been taken care of by at least one home health agency
3. I have never had direct experience with a home health agency but have heard about people's experiences with home health agencies.
4. I don't know much about home health agencies.

Q3. If you and the person you are taking care of/your family member have different preferences for the type of post-hospital care he or she receive, how will you decide where to receive post-hospital care?
1. I will respect my family member's preference and provide the care he/her needs during the recovery period.
2. I will discuss with my family member and jointly decide based on both of our preferences.
3. My family member will most likely follow my recommendation as I will be the primary person to provide care.

Q4. Are you retired?
1. Yes (if yes, stop here)
2. No (if no, go to Q5Q6)

Q5: How would you describe your work schedule flexibility? (Slider: from 1 to 5)

- Very flexible: I can choose to work from home or the office. I can easily change my workdays during the week. I can choose the number of hours I work during the week.
- Not flexible: I cannot choose to work from home or the office. I have a fixed workday schedule that cannot be changed easily. It is difficult for me to get vacation days.

Q6: How would you describe your job security? (Slider: from 1 to 5)

- Not secure: I am afraid to lose my job if I take a leave of absence for a period of time.
- Very secure: I am comfortable to take a leave of absence and not afraid to lose my job.

© 2024 Geng F et al. *JAMA Health Forum.*

eMethod 2. Technical details about the experimental design of our survey

In our study, we employed a labeled design for the discrete choice experiment because it allowed us to present the specific differences in receiving care services at a skilled nursing facility (SNF) or a home health agency (HHA) to the respondents more accurately.

Before finalizing the choice scenarios, we meticulously evaluated the survey's design and clarity. Initial tests were conducted within a group comprising 20 graduate students from a local college and 10 community members from a Massachusetts neighborhood. These initial tests facilitated cognitive interviews to assess participants' comprehension, ensuring that the questions were clear and captured the desired dimensions of post-acute care preferences. Participants, during this phase, specifically inquired about our survey instructions and the attributes of travel time and quality of care, signifying their active engagement and understanding of the survey's nuances. We enhanced the survey instructions and revised the experiment's attributes based on the feedback received.

Subsequently, we carried out two separate pilot studies on MTurk, Amazon's online task platform, with 25 participants each, to validate the practical relevance of the attribute range in our experiment. These participants varied in terms of education level, geographic location, and income. This was done to ensure the practical relevance of the attribute range in our experiment. The first pilot study utilized a fractional factorial design for a main effects model with no interactions, generated using Ngene 1.1.1 software. Guided by observations from this pilot, we adopted a D-efficient design for the subsequent pilot. This design incorporated prior assumptions about the sign of some parameters, including negative signs for attributes like travel time, daily time commitment from the primary family caregiver, patients' recovery period, and total out-of-pocket amount to exclude the dominated choices. Recognizing the potential variance in consumer value perceptions for SNFs and HHAs, we integrated alternative-specific parameter estimates for the overall quality rating of the facility/agency. This rigorous, multi-step validation process ensured the survey's robustness, addressing both clarity and comprehensiveness.

Additionally, for the choice of the participants, we would like to highlight that a significant proportion of our respondents—approximately 35%—have either stayed in a skilled nursing facility themselves or have cared for a family member who has, and approximately 49% have experience with home health agency care either personally or within their family. This suggests that a substantial portion of our participants have some familiarity with post-acute care experience.

Furthermore, our decision to include respondents without prior experience with post-acute care settings was motivated by a desire to understand societal perspectives, as many

individuals may face such decisions without previous exposure. While studies focusing solely on patients or caregivers with prior experience are valuable, our aim was to capture a broader societal viewpoint in this study.

Regarding the inclusion of hypothetical scenarios where a patient requires only 2 hours of caregiver help at home with home health services, our intention was to present a scenario where home health agencies could provide significant care to alleviate family burden even when patients choose to stay at home. To enhance realism, we ensured that in all the choice tasks, the caregiver time required for skilled nursing facilities was always less than that for home health agencies.

© 2024 Geng F et al. *JAMA Health Forum.*

eMethod 3. Model specifications

The random utility model provides the theoretical framework for DCEs. In this study, individual n is assumed to choose between two options, and will choose the option with higher utility. Thus, the main-effect utility model in our study can be defined as:

$V_{i,c} = ASC_{setting} * I_{setting,i,c} + \sum \beta_n * X_{n,i,c}$ (Deterministic Component)

$U_{i,c} = V_{i,c} + \varepsilon_{i,c}$,

Where $ASC_{setting}$ represents the alternative-specific constants for different care settings,
$I_{setting,i,c}$ is the binary indicator of nursing home or home in option c for a given individual i,
$X_{n,i,c}$ represents the value of the nth attribute in option c for a given individual i,
β_n represents the coefficient of the nth attribute,

Then, using the logit model, the probability of choosing an option c is defined as:

$$\Lambda(\text{Choice A} \mid X, Z) = \exp(V_i, A) / \exp(V_i, A) + \exp(V_i, B)$$

2) We subsequently examined the effects of various demographic and socio-economic determinants on intercept and attributes with significant deviations from a coefficient standard deviation of 0. These findings are based on the best-performing main-effect logit model, which encompassed:

a) Alternative-specific constant: post-acute care settings.

b) Attribute coefficients: Recovery durations, caregiver involvement, and SNF room types.

a) Regarding the alternative-specific constant for post-acute care settings, the deterministic component of the model for Exhibits 3 and 4 is formulated as:

$$V_{i,c} = ASC_{setting} * I_{setting,i,c} * Z_{m,i} + \sum \beta_n * X_{n,i,c}$$

$Z_{m,i}$ denotes the mth personal characteristics for a given individua l i,

© 2024 Geng F et al. *JAMA Health Forum.*

b) For interactions between personal characteristics and the kth attribute, the deterministic component of the model is defined as follows:

$$V_{i,c} = ASC_{setting} * I_{setting,i,c} + \sum_{n \neq k} \beta_n * X_{n,i,c} + \beta_k * X_{k,i,c} * Z_{m,i}$$

c) To assess the significance of impacts of specific demographic and socio-economic factors on both the intercept and attribute parameters, we implemented a two-step process:

Likelihood Ratio Test: Firstly, we used a likelihood ratio test to compare the more restrictive model against a complex model that presumes taste heterogeneity across different sub-populations. The hypothesis, which proposed the validity of the restricted model, was rejected in favor of the more intricate model.

Robust T-Test Analysis: For each parameter level (e.g., comparing coefficient estimates between employed and unemployed individuals), we tested the hypothesis that the coefficients were equivalent using a robust t-test. This robust t-test information was extracted from the "Correlation of Coefficients" section of the Biogeme model output. When the hypothesis was rejected, we concluded that the parameter estimates were statistically distinct between different population groups.

It is crucial to note that while our analyses did indicate significant influences from socio-economic factors like job security, employment status, prior experiences with HHAs and SNFs, and gender on the post-acute care settings for patients and caregivers.

© 2024 Geng F et al. *JAMA Health Forum.*

eTable 1. Additional Weighted Characteristics of Study Participants Who Completed the Survey (N=1,555), Representative of a National Sample.

Panel 1. Additional Weighted Characteristics of Study Participants Who Completed the Survey (N=1,555)

**Marital status**	
Married or living with a partner	62.9%
Separated	2.5%
Divorced	16.1%
Widowed	6.2%
Never married	12.2%
**Born in the U.S.**	91.3%
**Census Division related to the state of birth**	
New England	6.6%
Middle Atlantic	21.1%
East North Central	17.7%
West North Central	6.5%
South Atlantic	11.5%
East South Central	4.6%
West South Central	12.4%
Mountain	5.6%
Pacific	13.8%
**Census Division related to the state of residence**	
New England	6.5%
Middle Atlantic	15.5%
East North Central	11.9%
West North Central	4.6%
South Atlantic	17.1%
East South Central	4.2%
West South Central	13.4%
Mountain	9.9%
Pacific	16.8%

© 2024 Geng F et al. *JAMA Health Forum.*

**Employment status (some people belonged to more than 1 category)**	
Working now	48.4%
Unemployed and looking for work	1.8%
Temporarily laid off, on sick or other leave	2.4%
Disabled	10.6%
Retired	39.2%
Homemaker	7.0%
Other	4.2%
**Employment type**	
Work for someone else	83.3%
Self-employed	15.5%
Other	1.1%
**Health Insurance Type (some people belonged to more than 1 category)**	
Insurance through my or my spouse's/partner's employer/union	39.5%
Insurance through my parent's employer/union	0.7%
Retiree Insurance through my or my spouse's/partner's former employer/union	9.2%
Insurance through my state's or the federal health insurance exchange or marketplace	5.8%

© 2024 Geng F et al. *JAMA Health Forum.*

Self-pay insurance or private health insurance not through my state's or the federal health insurance exchange or marketplace	5.1%
Medicare	39.7%
Medi-Gap	9.7%
Medicaid	11.1%
Military health care (TRICARE/VA/CHAMP-VA)	5.4%
State-sponsored health insurance	1.7%
Other Government program	2.1%
Other	4.1%
**Wellbeing**	
1 Very dissatisfied	1.1%
2	0.8%
3	2.2%
4	2.5%
5	6.2%
6	8.2%
7	17.6%
8	25.3%
9	23.7%
10 Very satisfied	12.4%

Note: Authors' analysis of data from the online survey conducted with the American Life Panel. NOTES: Percentages might not add to 100 because of rounding. Statistics have been reweighted for national representativeness.

© 2024 Geng F et al. *JAMA Health Forum.*

Panel 2. A comparison on key demographic variables in our weighted sample and the data reported by the US Census Bureau for year 2022

Characteristic	Our weighted Sample (age 45+)	US Census Bureau for year 2022 (all age groups)
**Sex (Female)**	52.9%	50.4%
**Education**		
Less than or some high school with no diploma	7.3%	
High school graduate or equivalent	32.2%	High school graduate or higher 89.1% (age 25+)
Some college, no degree	15.0%	
Associate degree	10.2%	
Bachelor's and above	35.3%	Bachelor's degree or higher 34.3% (age 25+)
**Race**		
White/Caucasian	78.4%	75.5%
Black/African American	11.2%	13.6%
American Indian or Alaskan Native	1.7%	1.3%
Asian or Pacific Islander	2.2%	6.6%
Other	6.5%	3.0%
**Household size (persons per household)**	2.53	2.57

© 2024 Geng F et al. *JAMA Health Forum.*

eTable 2. Main-effect model performance comparison

Panel 1. Model comparison for patient perspective

Model	Nb. Of Parameters	Log likelihood	AIC	BIC	Simple size
M1 - Binary logit model	9	-3012.96	6043.91	6085.93	787
M2 - Binary logit model with alternative specific coefficients for quality of care in SNF and HHA	12	-3005.35	6043.71	6090.73	787
M3 - mixed logit model with random coefficients and variance	17	-2476.25	4986.5	5065.86	787
M4 - mixed logit model with alternative specific coefficients for quality of care in SNF and HHA	23	-2451.45	4948.9	5056.27	787
M5 - mixed logit model with alternative specific coefficients for quality of care in SNF and HHA, we only left the covariates as random variables where SD is statistically significant from 0	19	-2449.227	4936.454	5025.15	787

Panel 2. Main-effect model comparison for caregiver perspective

Model	Nb. Of Parameters	Log likelihood	AIC	BIC	Simple size
M1 - Binary logit model	9	-3164.76	6347.51	6389.31	768

© 2024 Geng F et al. *JAMA Health Forum.*

M2 - Binary logit model with alternative specific coefficients for quality of care in SNF and HHA	12	-3151.07	6326.15	6381.87	768
M3 - mixed logit model with random coefficients and variance	17	-2657.27	5348.54	5427.49	768
M4 - mixed logit model with alternative specific coefficients for quality of care in SNF and HHA	23	-2627.09	5300.18	5406.99	768
M5 - mixed logit model with alternative specific coefficients for quality of care in SNF and HHA, we only left the covariates as random variables where SD is statistically significant from 0	19	-2618.086	5274.171	5362.403	768

The reported results in the are based on the mixed logit model (M5) with alternative-specific coefficients for quality of care in SNF (Skilled Nursing Facilities) and HHA (Home Health Agencies). For covariates recovery time, caregiver time, below average quality of care for HHA and SNF, stay in private room, we only retained the covariates as random variables where the standard deviation (SD) is statistically significant from 0. This is under the assumption that:

1) Quality of care differs for respondents between SNF and home health settings. For instance, below-average quality of care in SNFs may be weighted more heavily by

© 2024 Geng F et al. *JAMA Health Forum.*

respondents due to the extended nature of their stay, thereby impacting their willingness to pay for lower/higher quality care.

2) There is heterogeneity in preferences among respondents for recovery time, caregiver time, below-average quality of care for both HHA and SNF, and staying in a private room.

© 2024 Geng F et al. *JAMA Health Forum.*

eTable 3. Regression analysis results for the best performing main-effect model - mixed logit model with alternative specific coefficients for quality of care and recovery time

Panel 1. Modeling results for patient perspective

Attributes	Model 1 - patient perspective	SD	WTP ($)	WTP per day($)	95% CI for point estimate WTP per day ($)
	**Coefficients**				
Location - HHA (relative to SNF)	**1.83*** **	**2.7*** **	**1841.05**	**58.08**	(45.32, 70.83)
Travel time (min)	**-0.0181*** **	0.0048	**-18.21**	-34.47	(-41.64, -27.29)
			(per 31.7 min)	(per hour)	(per hour)
Out of pocket cost ($)	**-0.000994*** **	--	--	--	--
Recovery Time (days)	**-0.0309*** **	**0.0463*** **	**-31.09**	**-31.09**	(-39.43, -22.74)
Caregiver Time (hour)	**-0.0481*** **	**0.17*** **	**-48.39**	**-1.53**	(-2.39, -0.66)
Quality of Care (relative to "average")					
below average_SNF	**-2.37*** **	0.614	**-2384.31**	**-75.21**	(-88.75, -61.68)
above average_SNF	**0.485*** **	--	**487.93**	**15.39**	(5.51, 25.27)
much above average_SNF	**0.682*** **	--	**686.12**	**21.64**	(10.15, 33.14)
below average_HHA	**-1.47*** **	**1.67*** **	**-1478.87**	**-46.65**	(-60.67, -32.64)
above average_HHA	0.0917	--	92.25	2.91	(-7.03, 12.85)
much above average_HHA	**0.838*** **	--	**843.06**	**26.59**	(14.55, 38.64)
Room type at SNF (compared to shared room)					
Stay in a private room	**0.685*** **	**1.37*** **	**689.13**	**21.74**	(13.11, 30.37)

© 2024 Geng F et al. *JAMA Health Forum.*

(mean: 31.7 days in the survey)					
Number of respondents	787				
Observations	6290				
Number of parameters	19				
AIC	5475.28				
BIC	5564.525				
loglikelihood(beta)	-2718.914				

SOURCE: Authors' analysis of data from the online survey conducted with the American Life Panel. NOTE: Model output from alternative-specific model. Statistically significant levels: *** $p<0.01$, ** $p<0.05$, * $p<0.1$.

© 2024 Geng F et al. *JAMA Health Forum.*

Panel 2. Modeling results for caregiver perspective

**Attributes**	**Model 2 - caregiver perspective**	**SD**	**WTP ($)**	**WTP per day($)**	**95% CI for point estimate WTP per day ($)**
	**Coefficients**				
Location - HHA (relative to SNF)	**0.973*** **	**2.16*** **	**1443.62**	**45.54**	(31.09, 59.99)
Travel time (min)	**-0.0173*** **	**0.00997** **	**-25.67**	**-48.58**	(-58.71, -38.46)
			(per 31.7 min)	(per hour)	
Out of pocket cost ($)	**-0.000674*** **	--	--	--	--
Recovery Time (days)	**-0.0205*** **	**0.0308*** **	**-30.42**	**-30.42**	(-40.13, -20.70)
Caregiver Time (hour)	**-0.121*** **	**0.139*** **	**-179.53**	**-5.66**	(-6.82, -4.50)
Quality of Care (relative to "average")					
below average_SNF	**-1.69*** **	0.685	**-2507.42**	**-79.10**	(-94.91, -63.29)
above average_SNF	**0.511*** **	--	**758.16**	**23.92**	(11.68, 36.16)
much above average_SNF	**1.00*** **	--	**1483.68**	**46.80**	(31.49, 62.11)
below average_HHA	**-0.803*** **	1.281	**-1191.39**	**-37.58**	(-53.12, -22.05)
above average_HHA	0.263*	--	390.21	12.31	(-0.97, 25.59)
much above average_HHA	**1.08*** **	--	**1602.37**	**50.55**	(35.49, 65.61)
Room type at SNF (compared to shared room)					
Stay in a private room	**0.58*** **	**0.831** **	**860.53**	**27.15**	(17.03, 37.26)
(mean: 31.7 days in the survey)					
Number of respondents	768				

© 2024 Geng F et al. *JAMA Health Forum.*

Observations	6142				
Number of parameters	19				
AIC	6013.65				
BIC	6101.882				
loglikelihood(beta)	-2987.825				

SOURCE: Authors' analysis of data from the online survey conducted with the American Life Panel. NOTE: Model output from alternative-specific model. Statistically significant levels: *** $p<0.01$, ** $p<0.05$, * $p<0.1$.

© 2024 Geng F et al. *JAMA Health Forum.*

eMethod 4. Calculation of Willingness-to-Pay (WTP) Estimates and the underlying assumptions for WTP estimates

1） Equation for Deriving WTP from Coefficients:
WTP=coefficient of the attribute in question / coefficient of out-of-pocket cost

2) Rationale for Utilizing WTP Per Day:
In the survey, respondents were presented with potential recovery periods of 20, 30, or 45 days. It's reasonable to posit that WTP concerning certain attributes might fluctuate based on the recovery period. For instance, the value assigned to a 1-hour reduction in travel time over a 30-day recovery period might surpass that for a 20-day period. To standardize these variations and offer a coherent frame of reference, WTP values were converted to a daily basis.

Furthermore, we've operated under the assumption that WTP for these attributes is linearly related to the length of the recovery period. For clarity, if the WTP for a 1-hour reduction in travel time during a 30-day recovery is considered, it's projected to be 50% greater than that during a 20-day period. Thus, the daily WTP is derived by dividing the total WTP by the weighted average recovery period (approximately 31.7 days in our data).

3) WTP Estimations for different attributes:

- Recovery Time: The presented WTP inherently refers to the value for a one-day reduction in the recovery period.
- Travel and Caregiver Time: Daily WTP refers to the value ascribed to saving 1 hour each day.
- Other Attributes: Daily WTP reflects the value placed on the attributes for one singular day.

This approach was used to present WTP estimates on a daily basis, providing clarity and enabling comparisons, given that the cost attribute encapsulated the total expenditure, not a daily figure.

© 2024 Geng F et al. *JAMA Health Forum.*

eFigure 1. Participant Engagement: Responses to Survey Interest Query

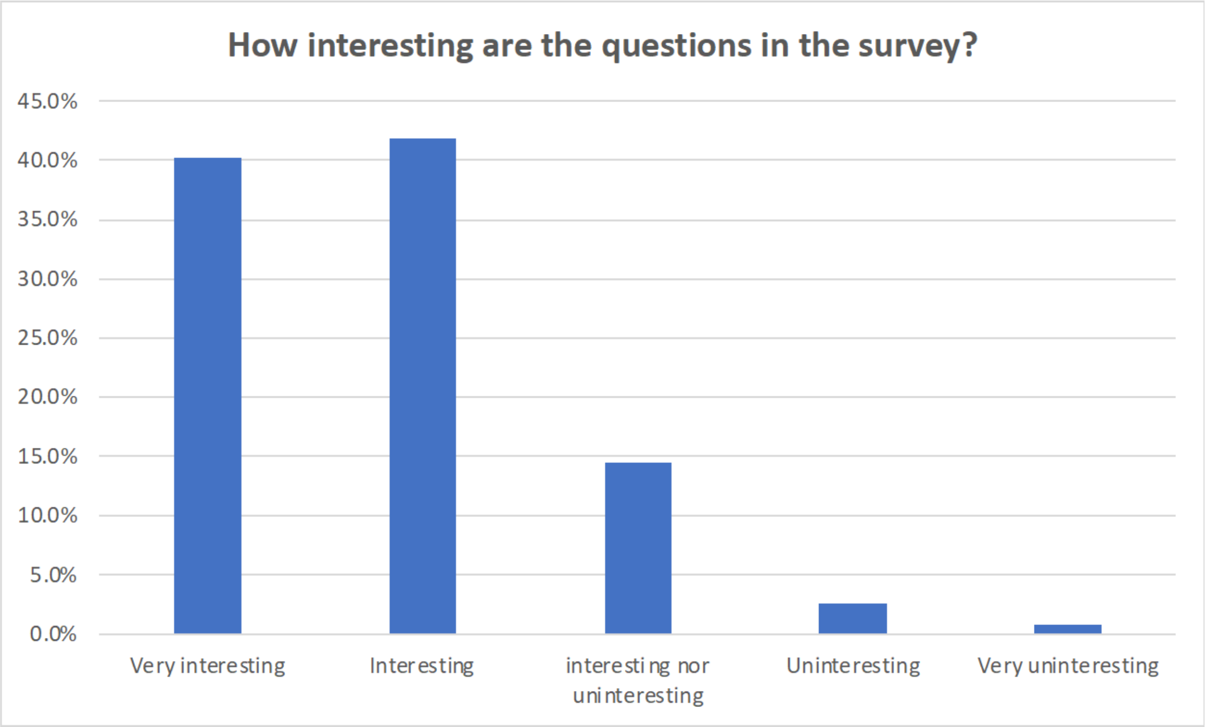

Note: Sample size N=1,555.

© 2024 Geng F et al. *JAMA Health Forum.*

eTable 4. Descriptive Statistics of Respondent Characteristics, Comparing Unweighted Data Between Survey Completers and Non-Completers.

	Complete	Incomplete
Characteristic	Percent or mean	Percent or mean
**Sex (Female)**	55.2%	55.6%
**Age**	64.3	61.6
**Education**		
Less than or some high school with no diploma	2.3%	2.5%
High school graduate or equivalent	11.4%	12.5%
Some college, no degree	19.2%	24.9%
Associate's degree	13.1%	15.3%
Bachelor's and above	54.0%	44.8%
**Race**		
White/Caucasian	84.2%	74.3%
Black/African American	8.8%	14.9%
American Indian or Alaskan Native	1.3%	1.0%
Asian or Pacific Islander	1.9%	3.1%
Other	3.8%	6.7%
**Household size**	2.17	2.41
**House Type**		
A one-family house	78.2%	77.6%
A building with apartments	18.3%	17.6%
A mobile home, boat, RV, van, etc.	3.5%	4.0%
**Annual Household income**		
Less than $19,999	8.9%	11.1%
$20,000 to $39,999	17.1%	18.0%
$40,000 to $74,999	29.3%	28.5%
$75,000 to $124,999	22.8%	22.8%
$125,000 to $199,999	14.4%	11.3%
$200,000 or more	7.5%	8.0%
**Urban/Rural**		
Small to midsize city or Large city, 50K+ population	73.8%	79.7%
Rural or Small Town, population under 50K	26.2%	20.3%
**General Health**		

© 2024 Geng F et al. *JAMA Health Forum.*

Excellent	11.6%	7.1%
Very Good	39.8%	36.0%
Good	33.8%	31.6%
Fair	12.5%	12.5%
Poor	2.3%	2.1%
**Total number of observations**	1555	522

© 2024 Geng F et al. *JAMA Health Forum.*

eTable 5. Prior experience with skilled nursing facilities and home health care and post-acute care decision-making

	Patients' perspective	Caregivers' perspective
Total number of respondents	787	768
Previous experience with nursing homes		
I have stayed in at least one skilled nursing facility.	2.8%	3.5%
I have taken care of at least one family member who has stay in nursing homes	33.1%	29.4%
I have visited at least one skilled nursing facility.	30.7%	37.5%
I have never had direct experience with a skilled nursing facility.	13.6%	14.6%
I don’t know much about skilled nursing facilities.	19.7%	15.0%
**Previous experience with home health care**		
I have been taken care of by at least one home health agency.	9.2%	8.1%
I have taken care of at least one family member who has been taken care by home health agency.	39.4%	40.5%
I have never had direct experience with a home health agency.	29.9%	32.0%
I don’t know much about home health agencies.	21.5%	19.5%
**If you and your primary caregiver have different preferences for the type of post-hospital care you receive, how will you decide where to receive post-hospital care?**		

© 2024 Geng F et al. *JAMA Health Forum.*

Decide mostly based on patients' preference and the caregiver will respect his/her decision.	29.2%	33.3%
Discussion between the patient and the primary caregiver and decide based on their joint preferences.	59.8%	55.3%
Decide mostly based on the caregiver's preference as the patients will rely on him/her to provide the needed care.	11.0%	11.4%

Note: Authors' analysis of data from the online survey conducted with the American Life Panel. Statistics have been reweighted for national representativeness.

© 2024 Geng F et al. *JAMA Health Forum.*
