# Supplementary material for: Preferences for Postacute Care at Home vs Facilities
Source: JAMA Health Forum. 2024 Apr 26;5(4):e240678. doi: 10.1001/jamahealthforum.2024.0678 (PMC11065156; doi:10.1001/jamahealthforum.2024.0678)
Supplement: Supplement 2. — Data sharing statement [file jamahealthforum-e240678-s002.pdf]

Data Sharing Statement

Geng. Preferences for Postacute Care at Home vs Facilities. *JAMA Health Forum*. Published April 26, 2024. doi:10.1001/jamahealthforum.2024.0678

Data
Data available: Yes
Data types: Deidentified participant data
How to access data: https://www.rand.org/research/data/alp/data-access.html
When available: With publication

Supporting Documents
Document types: None

Additional Information
Who can access the data: Researchers whose proposed use of the data has been approved
Types of analyses: For research purpose
Mechanisms of data availability: With a signed data access agreement on the Data Access website.
Any additional restrictions: Please cite this paper for the method of details on the survey design and data collection methodology.
